# Is additional 5-day vasoactive drug therapy necessary for acute variceal bleeding after successful endoscopic hemostasis? A systematic review and meta-analysis

**DOI:** 10.1097/MD.0000000000012826

**Published:** 2018-10-12

**Authors:** Pengguang Yan, Xiao Tian, Jingnan Li

**Affiliations:** a Peking Union Medical College, Dongdan Santiao; b Peking Union Medical College Hospital, Shuaiguyuan, Beijing, China.

**Keywords:** endoscopic hemostasis, esophageal variceal bleeding, optimal duration, vasoactive drugs

## Abstract

**Background:**

Vasoactive drugs and endoscopic therapy have been widely used in the management of acute variceal bleeding of cirrhosis patients. The current standard regimen of vasoactive drugs is in combination with endoscopic therapy and continues for up to 5 days; however, the necessity of vasoactive drugs after endoscopic hemostasis was still controversial. Therefore, we conducted a systematic review and meta-analysis to evaluate the efficacy and optimal duration of adjuvant vasoactive drugs after hemorrhage control by endoscopic therapy.

**Methods:**

A search was conducted of PubMed, EMBASE, and Cochrane Library databases until June, 2018. Lan DeMets sequential monitoring boundary was constructed to assess the reliability and conclusiveness of our major results.

**Results:**

Seven studies (639 patients) and 4 studies (435 patients) were included in the analyses to evaluate the efficacy and optimal duration of adjuvant vasoactive drugs therapy, respectively. Our analyses showed that adjuvant vasoactive drugs facilitated endoscopic hemostasis and reduced very early re-bleeding rate both in sclerotherapy (risk ratio [RR] 0.51, 95% confidence interval [CI] 0.34–0.78, *P* = .23, *I*^2^ = 31%) and band ligation (RR 0.48, 95% CI 0.27–0.83, *P* = .07, *I*^2^ = 62%). However, the 3 to 5-day therapy duration was not superior to a shorter course in very early re-bleeding rate and mortality rate in 42 days (RR 1.77, 95% CI 0.64–4.89, *P* = .70, *I*^2^ = 0%; RR 0.95, 95% CI 0.43–2.13, *P* = .81, *I*^2^ = 0%, respectively).

**Conclusion:**

Additional 5-day vasoactive drug after endoscopic hemostasis may significantly ameliorate very early re-bleeding rate, However, the 3 to 5 days’ adjuvant regimen was not superior to a shorter course.

## 1 Introduction

Acute variceal bleeding (AVB) is the most life-threatening complication in patients with liver cirrhosis due to portal hypertension; even with current standards of treatment, mortality associated with esophageal variceal bleeding typically reaches 20%.^[1,2]^ In Child-Pugh C patients, mortality still remains >30%.^[3]^

Vasoactive drugs are effective in variceal bleeding control due to the efficacy in reducing the pressure in both the portal vein and varices.^[1]^ They have been recognized as the first-line treatment, associated with a significant increase in hemostasis and lower risk of 7-day mortality.^[4]^ Current consensus suggested vasoactive drugs should be started as soon as possible, before the diagnostic endoscopy,^[5]^ since adjuvant pharmacologic treatment is more effective than endoscopic therapy alone.^[6]^ Indeed, early administration of vasoactive drugs could ease the endoscopic procedure and ameliorate initial hemorrhage control,^[7–10]^ while the efficacy of vasoactive drugs after endoscopic hemostasis is controversial partly due to the advancement in endoscopic techniques and inadequate understanding of the mechanism and pharmacokinetics of vasoactive drugs in portal pressure management.

Some randomized trials have shown that adjuvant drug infusion after endoscopic therapy (sclerotherapy or variceal ligation) does not offer any advantages in the prevention of very early re-bleeding or reducing mortality.^[11–14]^ Conversely, other studies have concluded that vasoactive drugs are superior to placebo in postendoscopic AVB treatment.^[15–17]^ Moreover, several publications failed to demonstrate adjuvant 5-day vasoactive drug treatment was superior to a shorter course if AVB was successfully controlled by endoscopic therapy; a shorter course was associated with cost savings and shorter hospital stay.^[18–20]^ The purpose of our work was to conduct a systematic review and meta-analysis to evaluate the efficacy and optimal duration of vasoactive drugs after successful hemorrhage control by endoscopic therapy.

## 2 Methods

### 2.1 Search strategy

Two investigators (PY and XT) independently performed a systematic literature retrieval using electronic databases including PubMed, EMBASE, and Cochrane Library databases. The retrieval was finished in June, 2018 using the search strategy that included the terms for “vasoactive drug,” “endoscopic therapy,” and “esophageal variceal hemorrhage.” We used the following Mesh terms and words when searching in PubMed: ((Somatostatin) OR (Octreotide) OR (lanreotide) OR (pasireotide) OR (vapreotide) OR (vasoactive) (terlipressin) OR (vasopressin)) AND ((endoscop^∗^) OR (endoscopic variceal ligation) OR “sclerotherapy”[MeSH Terms] OR sclerotherapy[Text Word]) AND ((haemorrhage^∗^) OR (hemorrhage^∗^) OR (bleed^∗^)) AND (varice^∗^)) OR (hematemesis[Title/Abstract]) OR (melena[Title/Abstract]) OR (“Esophageal and Gastric Varices”[Mesh]) OR (“Hematemesis”[Mesh]) OR (“Melena”[Mesh])) AND ((randomized controlled trial[Publication Type]) OR (controlled clinical trial[Publication Type]) OR (random^∗^[Title/Abstract]) OR (trial[Title/Abstract]) OR (placebo[Title/Abstract]) OR (group[Title/Abstract])). Two investigators independently assessed the eligibility based on titles and abstracts, and retrieved the full texts for further extraction of the study details. No language limitation was applied.

### 2.2 Criteria for inclusion and exclusion

Inclusion studies were randomized controlled trials (RCTs) whose enrolled patients had active esophageal variceal bleeding or signs of recent bleeding with the same major pre-endoscopic management (with or without vasoactive drugs infusion) that were treated with somatostatin, octreotide, or terlipressin, and that reported the rate of re-bleeding and mortality. Studies were excluded if they were not RCTs, enrolled patients with gastric varices, gastrointestinal ulcer, or other possible sources of bleeding other than esophageal varices, and that republished studies or the full texts were not available were also excluded.

### 2.3 Data extraction

PY and XT extracted data independently using a preplanned extraction form in an Excel spreadsheet. Data were extracted from the included original RCTs. Disagreements were resolved by discussion between the 2 authors. The extracted data included: title of the study, name of the first author, year of study, year of publication, country of origin, patient characteristics, sample size, and below-mentioned outcomes.

### 2.4 Assessment of risk of bias in included studies

According to The Cochrane Collaboration criteria,^[21]^ we assessed the risk of trials based on random sequence generation, allocation concealment, blinding of participants and personnel, blinding of outcome assessment, incomplete outcome data, selective reporting, and other bias.

### 2.5 Outcome measurements

The primary outcomes were very early re-bleeding (re-bleeding in 5 days after endoscopic therapy), 5 and 42-day mortality rate, and adverse effects. Re-bleeding was defined as failure of hemostasis since hemorrhage control after endoscopic therapy.

### 2.6 Statistical analysis

Data analysis was performed using Review Manager 5.2 software from the Cochrane Collaboration (London, UK). Heterogeneity among the analyzed studies was assessed by the *I*^2^ statistic. A value of *I*^2^ of 0% to 25% represents insignificant heterogeneity, greater than 25% but less than or equal to 50% represents low heterogeneity, greater than 50% but less than or equal to 75% represents moderate heterogeneity, and greater than 75% represents high heterogeneity. A fixed-effects model or random-effects model was used selectively based on the level of heterogeneity for the estimation of the risk ratio (RR) and the respective 95% confidence intervals (CIs) for the analyzed outcomes.

Trial sequential analysis was performed to evaluate our major positive results. The sample size needed for a conclusive meta-analysis is supposed to be no smaller than a single optimally powered randomized control trial, so the optimal sample size required for our meta-analysis was calculated and used for the construction of a Lan DeMets sequential monitoring boundary to assess the reliability and conclusiveness of results.

### 2.7 Ethical approval

As it is a meta-analysis of the previous works of literature, approval of the ethics committee was not required.

## 3 Results

**Figure 1:**
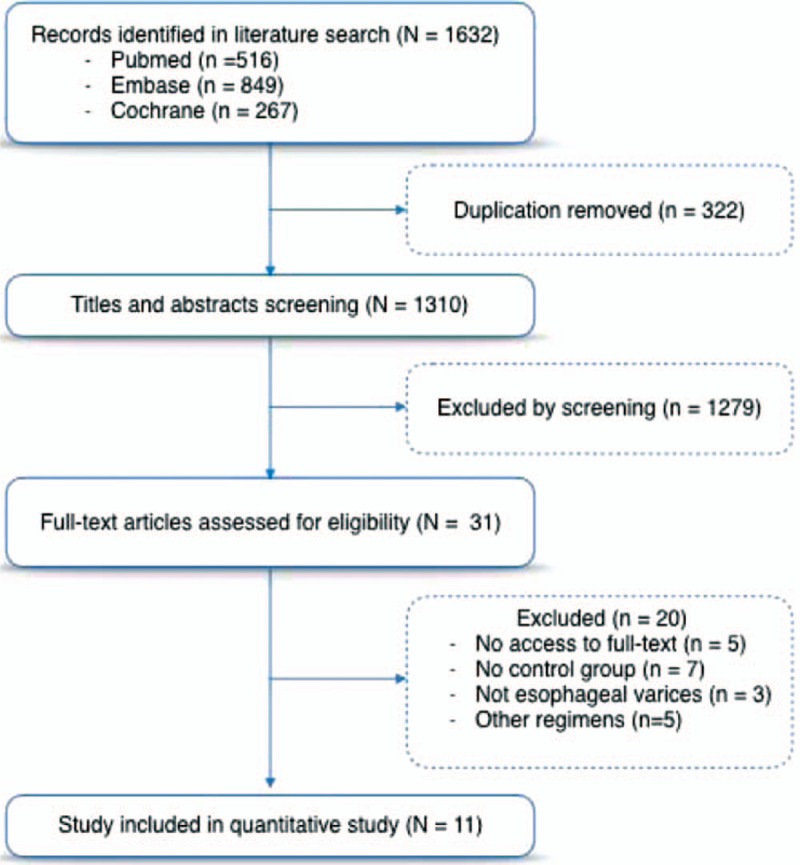
Literature review process.

We identified 1632 references through the electronic searches (EMBASE 849, Cochrane 267, PubMed 516). Among them, 322 duplicates and 1279 clearly irrelevant references were excluded based on the title and abstract. The table characteristics of included studies provide details on the characteristics of the included studies. We provisionally selected a total of 31 studies as potentially fulfilling the inclusion criteria. Eleven studies were included after full-text review and 20 studies were excluded. Eleven randomized controlled clinical trial studies^[11–14,16–19,22–24]^ matched the selection criteria finally. The detailed process of the literature search is depicted in Fig. 1. The characteristics of the patients in each study are shown in Table 1.

**Table 1:**
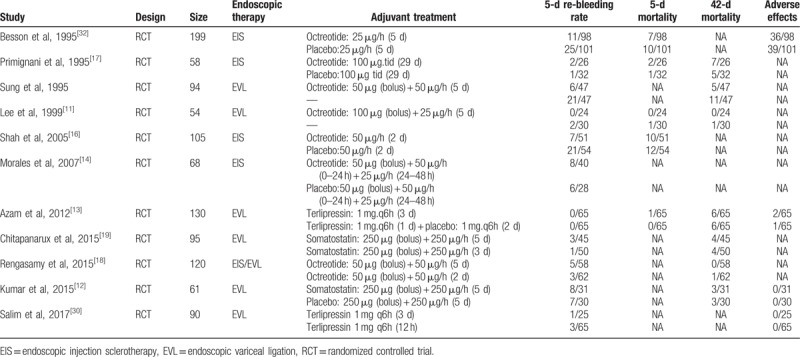
Main characteristics of the studies included in this meta-analysis.

### 3.1 Risk of bias in included studies

Considering the predefined modified Jadad criteria (allocation concealment, blinding of outcome assessor, and incomplete outcome data) to assess the overall risk of bias of a study, we judged 9 out of 11 (81.8%) trials at low risk of bias,^[12–14,16–19,23,24]^ and 2 of 9 (22.2%) trials at high risk of bias.^[11,22]^

### 3.2 Outcomes

The first analysis involving 639 patients in 7 trials evaluated the efficacy of adjuvant vasoactive drugs after endoscopic hemostasis. Four and 3 trials were enrolled in the endoscopic injection sclerotherapy (EIS) and endoscopic injection sclerotherapy (EVL) subgroups, respectively, according to different endoscopic hemostasis techniques.

### 3.3 Very early re-bleeding

**Figure 2:**
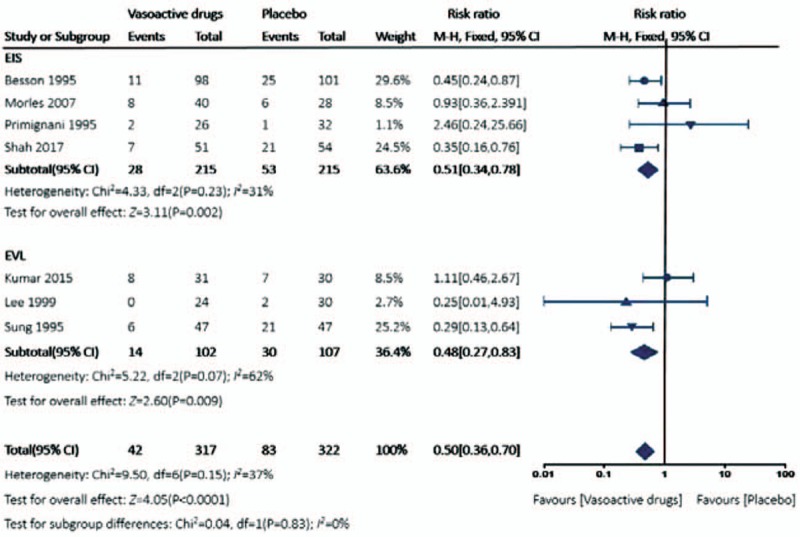
Forest plot for very early re-bleeding rate comparing adjuvant vasoactive drugs versus placebo.

**Figure 3:**
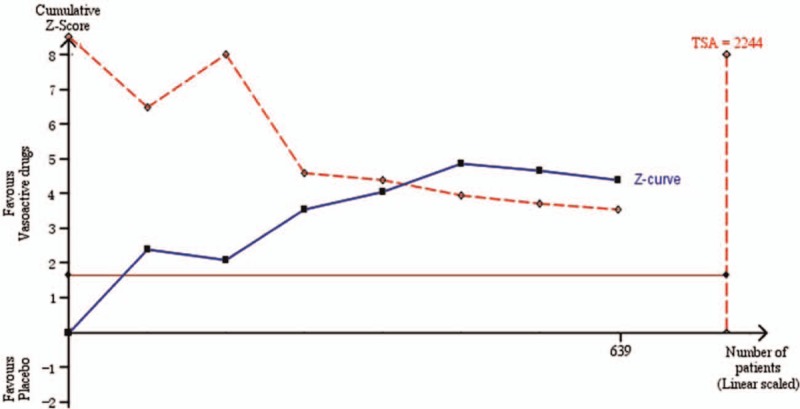
Cumulative meta-analysis assessing the efficacy of adjuvant vasoactive drugs on patients with acute variceal bleeding after endoscopic hemostasis.

The risk of re-bleeding after adjuvant vasoactive drugs therapy was significantly lower than the control group by the fixed-effect model (RR 0.50, 95% CI 0.36–0.70) The overall heterogeneity was acceptable (*P* = .15, *I*^2^ = 37%) (Fig. 2), and the crossed boundary in Lan-DeMets sequential monitoring boundary that assumes a 25.8% control event rate and a 25% relative risk reduction with 80% power and a 1-sided upper α = 0.05 indicated the significant difference was conclusive and reliable (Fig. 3). A similar trend was achieved in both EIS subgroup (RR 0.51, 95% CI 0.34–0.78, *P* = .23, *I*^2^ = 31%) and EVL subgroup (RR 0.48, 95% CI 0.27–0.83, *P* = .07, *I*^2^ = 62%). The heterogeneity in EVL subgroup was relatively acceptable, potentially explained by differences in included criteria, medications, and endoscopic devices.

### 3.4 5-day mortality rate

**Figure 4:**
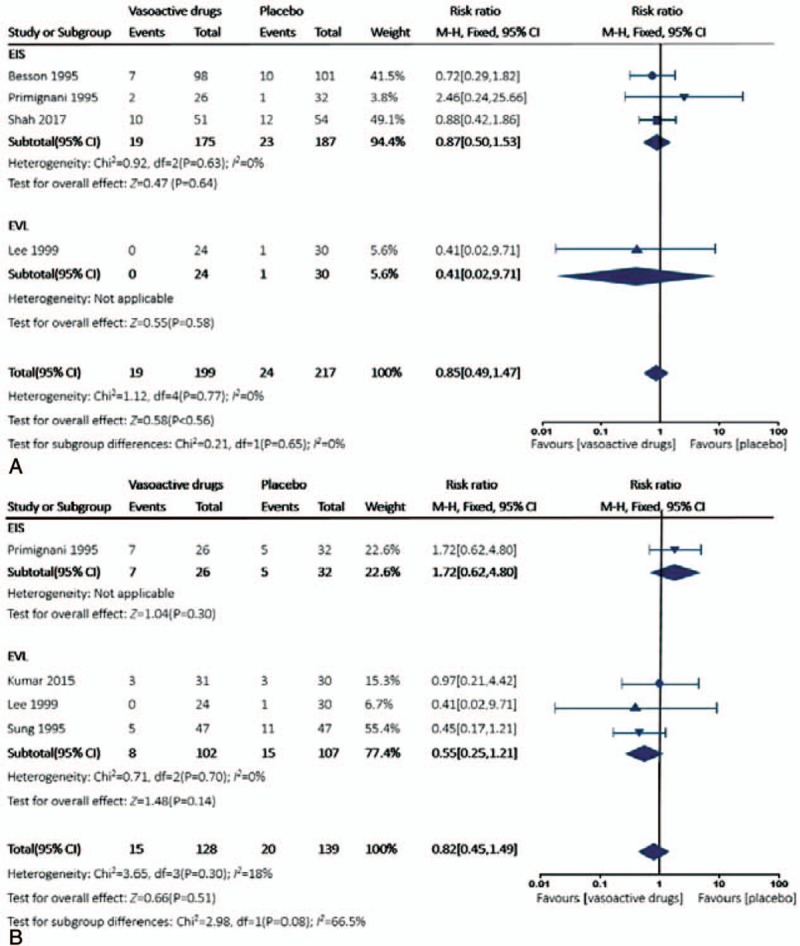
Forest plot for all-cause mortality comparing adjuvant vasoactive drugs versus placebo. (A) 5-day mortality; (B) 42-day mortality.

Compared with control group, adjuvant use of vasoactive drugs marginally reduced 5-day mortality (RR 0.85, 95% CI 0.49–1.47). Fixed-effect model was used since the heterogeneity was insignificant (*P* = .77, *I*^2^ = 0%). Similarly, in subgroup analysis, there was no significant difference in the 5-day mortality in both EIS subgroup (RR 0.87, 95% CI 0.50–1.53, *P* = .63, *I*^2^ = 0%) and EVL subgroup (RR 0.41, 95% CI 0.02–9.71) (Fig. 4).

### 3.5 42-day mortality rate

There was no significant difference in the 42-day mortality between the 2 groups by fixed-effect model (RR 0.82, 95% CI 0.45–1.49, *P* = .30, *I*^2^ = 18%); in addition, the difference was not remarkable either in EIS subgroup (RR 1.72, 95% CI 0.62–4.80) or in EVL subgroup (RR 0.55, 95% CI 0.25–1.21, *P* = .70, *I*^2^ = 0%) (Fig. 4).

### 3.6 3 to 5-day regimen versus a shorter course

The second analysis involving 435 patients in 4 trials evaluated the optimal duration of adjuvant vasoactive drugs after endoscopic hemostasis. All patients except 22 in the study by Rengasamy et al^[18]^ accepted band ligation hemostasis, and sclerotherapy was performed in the remaining patients.

**Figure 5:**
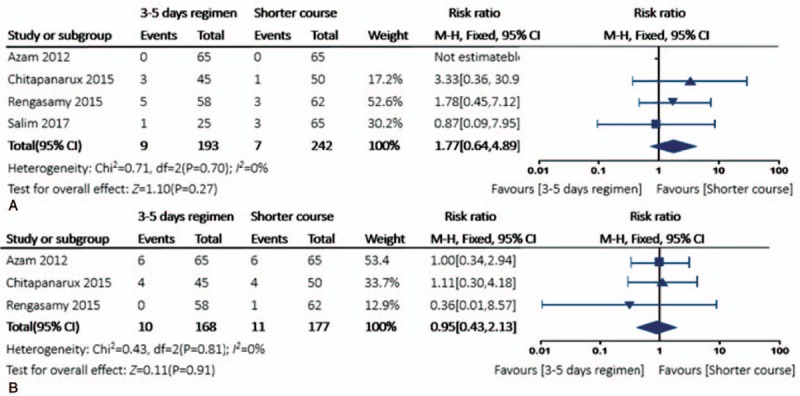
Forest plot for the re-bleeding rate in 5 and 42-day mortality comparing 3 to 5 days’ adjuvant vasoactive drug therapy versus a shorter course. (A) Very early re-bleeding rate; (B) 42-day mortality.

There was no significant difference in the risk of 42-day mortality rate (RR 0.95, 95% CI 0.43–2.13, *P* = .81, *I*^2^ = 0%) comparing a 3 to 5-day vasoactive drugs regimen with a shorter course. In the evaluation of very early re-bleeding rate, a shorter course was even better (RR 1.77, 95% CI 0.64–4.89, *P* = .70, *I*^2^ = 0%) (Fig. 5), although it was not statistically significant.

## 4 Discussion

Terlipressin, somatostatin, and octreotide are the recommended vasoactive drugs for the management of acute variceal bleeding, the comparisons between which failed to demonstrate a significant difference for the major outcomes,^[4]^ whereas somatostatin and octreotide were associated with better safety profiles.^[25]^ It is commonly suggested the duration of pharmacology therapy should last for 5 days after initial hemostasis, because this time span encompasses the greatest risk of bleeding,^[26]^ and it gradually became the conventional treatment duration in several RCTs. A systematic review suggested long-term treatment for 5 days for its successful management in hemostasis, prevention of very early re-bleeding and low incidence of severe side-effects^[27]^; hence, the current consensus recommended vasoactive drugs should be used in combination with endoscopic therapy and continued for up to 5 days (1a; A).^[5]^ However, there is no valid evidence on the efficacy of additional vasoactive drugs after successful endoscopic therapy,^[28]^ especially the optimal duration of the vasoactive regimen. In all, 639 patients were randomized in the 7 trials that evaluated the efficacy of postendoscopic treatment of vasoactive drugs. The adjuvant pharmacologic treatment significantly improved very early re-bleeding rate after endoscopic hemostasis. Although the enrolled patients are much smaller than our calculated optimal information size (2244 patients, based on the 25.8% control event rate in current trails), the constructed Lan-DeMets sequential monitoring boundary showed there was a crossover, indicating that the cumulative evidence is conclusive. Vasoactive drugs and endoscopic treatment have different mechanisms in variceal bleeding control, direct hemostasis under endoscope or reduction in hepatic venous pressure gradient by vasoactive drugs. There is an immediate increase in portal pressure after sclerotherapy and it lasts for at least 5 days^[29]^; hence the vasoactive drugs may counterbalance this rising pressure, ameliorating the risk of very early re-bleeding. The results were similar in the band ligation subgroup in which 3 studies including a total of 209 patients were enrolled in the subgroup analysis, which was far less than the calculated optimal information size. We must interpret our results with caution due to small sample size.

Moreover, the overall incidence of very early re-bleeding (28.04%) in placebo group was higher than the documented risks in the recent studies,^[30]^ partially because most of them were taken in the 1990s; the introduction of multiband devices in recent years may be associated with better short-term outcomes, hence the 5-day adjuvant vasoactive drug after endoscopic hemostasis seems invalidated.

The optimal adjuvant vasoactive drugs regimen analysis indicated the 3 to 5-day duration was not associated with a better profile than a shorter course. Because among the widely used vasoactive drugs, octreotide was in correlation with rapid desensitization and/or tachyphylaxis, although a marked and transient decrease in portal pressure could be observed after the initial bolus, a continuous infusion did not maintain or prolong its effects.^[31]^ Conversely, 5-day adjuvant vasoactive drug therapy was associated with a longer hospital stay, more packed red cell transfusion requirement, and higher expenses.^[18–20]^ Although vasoactive drugs are relatively safe and exert fewer adverse effects, especially with octreotide, taken cost-effectiveness into clinical strategy, a less than 3-day adjuvant vasoactive drugs regimen after endoscopic hemostasis should be considered.

Although most of the included studies were of high quality, there were several limitations in this meta-analysis. First, we could not avoid publication bias completely as there were a limited number of included studies. Second, although the low heterogeneity existed among the sclerotherapy subgroup could be explained by different treatment regimens, characteristics of involved patients, and different endoscope devices, it also affected the reliability of our results. Third, the sample size enrolled in this study was rather small, notwithstanding trial sequential analysis was performed; we need to interpret the results with caution. Finally, we did not evaluate several second outcomes such as hospital stay and amount of transfusion, because original data were unavailable in most studies.

## 5 Conclusions

In conclusion, this meta-analysis suggested that adjuvant vasoactive drugs after endoscopic hemostasis may significantly ameliorate re-bleeding rate, but the efficacy of adjuvant 5-day vasoactive drugs after successful bind ligation was not superior to a shorter course. Nevertheless, these findings were based on varied patient characteristics and small sample size, predictive scale was supposed to be established involving Child-Pugh score, HVPG, and endoscopic score to evaluate the risk of re-bleeding and mortality in acute variceal bleeding patients, and further randomized clinical trials need to be conducted to validate the efficacy and optimal duration of additional vasoactive drugs use after successful endoscopic hemostasis.

## Author contributions

**Conceptualization:** Pengguang Yan, Xiao Tian, Jingnan Li.

**Data curation:** Pengguang Yan, Xiao Tian.

**Formal analysis:** Pengguang Yan, Xiao Tian.

**Investigation:** Pengguang Yan.

**Project administration:** Pengguang Yan.

**Validation:** Pengguang Yan, Xiao Tian.

**Writing – original draft:** Pengguang Yan, Xiao Tian.

**Software:** Xiao Tian.

**Methodology:** Jingnan Li.

**Writing – review & editing:** Jingnan Li.
